# Effect of Arthroscopic Acromioplasty on the Isometric Abduction Strength

**DOI:** 10.7759/cureus.59426

**Published:** 2024-04-30

**Authors:** Ahmed M Heydar, Görkem Kıyak

**Affiliations:** 1 Orthopedics and Traumatology, Bahçelievler Memorial Hospital, Istanbul, TUR; 2 Orthopedics and Traumatology, Academic Hospital, Istanbul, TUR

**Keywords:** deltoid muscle, abduction strength, impingement, acromioplasty, arthroscopy, acromion

## Abstract

Introduction

Acromioplasty is a widely performed procedure for various rotator cuff pathologies with good outcomes and high patient satisfaction. However, few studies have focused on its potential complications. Previous cadaveric studies have demonstrated that a considerable portion of the deltoid muscle is detached from its acromial origin following arthroscopic acromioplasty, but the clinical relevance of this muscle detachment has not been investigated. The goal of our research was to examine the influence of arthroscopic acromioplasty on abduction strength and to assess whether acromial anatomy plays a role in any potential effect.

Methods

From a preliminary sample of 87 individuals who were diagnosed with isolated impingement syndrome and underwent arthroscopic acromioplasty, 74 patients who met the inclusion criteria were ultimately included in the study. The patients were divided into two groups according to their acromion morphology: Bigliani type 2 (33 patients) and type 3 (41 patients). The isometric abduction strength of the two groups was measured by a handheld dynamometer (Isobex®; Cursor AG, Berne, Switzerland) at different abduction angles preoperatively and at the first, third, and sixth months following surgery and was statistically compared.

Results

Both groups showed reduced abduction strength postoperatively; however, the strength of abduction in the Bigliani type 3 group returned to near preoperative values in the third month. Although increased mean abduction strength was recorded at 30° abduction in the sixth month, this difference was not statistically significant (p=0.78). In the Bigliani type 2 group, compared with those in the sixth-month group, the preoperative abduction strength decreased from 8.32 kg to 6.0 kg (p = 0.047), 6.57 kg to 5.15 (p = 0.025), and 6.1 kg to 4.56 kg (p = 0.006) at 30, 60, and 90° abduction, respectively.

Conclusions

Arthroscopic acromioplasty decreased isometric abduction strength in patients with a Bigliani type 2 acromion. Patients should be counseled about this loss, which might be especially important for professional athletes and heavy manual workers.

## Introduction

Acromioplasty is a well-described technique applied for various rotator cuff pathologies either alone or with concomitant rotator cuff repair. It was first described in 1972 for the treatment of chronic impingement syndrome by Neer, who blamed the acromial morphology for rotator cuff tendinopathy and subsequent tearing [1]. Subsequently, acromion has been classified according to morphology into three types and correlated hooked acromion (type 3) with rotator cuff pathologies [2]. Since then, several modifications to acromioplasty have been proposed, and all of them insist on the importance of the integrity of the deltoid attachment to the acromion, especially the anterior component [3]. Thereafter, Hohmann described a novel procedure, arthroscopic subacromial decompression, to replace open acromioplasty, and he reported promising results [4]. The procedure consisted of arthroscopic bone resection of the anterior acromion undersurface, coracoacromial ligament release, and bursal debridement. The most important advantage of the arthroscopic approach is the theoretical preservation of the integrity of the deltoid origin, in addition to improved cosmesis of the surgical scar and accelerated recovery [5]. Ultimately, arthroscopic acromioplasty has taken the place of open acromioplasty as the preferred method for decompressing the subacromial space [6].

Perhaps good outcomes and high patient satisfaction with arthroscopic acromioplasty [4,7] were the reasons behind the increased popularity and rapid increase in the number of performed acromioplasties [8]. However, limited studies have focused on potential complications that may arise following arthroscopic acromioplasty. Few studies have investigated the effect of arthroscopic acromioplasty on the acromial attachment of the deltoid origin. Torpey et al. [9] demonstrated that a considerable portion of deltoid anterior and lateral fiber attachments dehisced following arthroscopic acromioplasty when 4-6 mm of the acromion undersurface was removed. However, they could not find any correlation between acromial morphology and the number of dehisced fibers. Similarly, Green et al. [10] reported a possible extensive effect of arthroscopic acromioplasty on the deltoid origin. Moreover, they found that the greater the acromial spurring was, the less the deltoid origin was detached, and acromioplasty to a thin and flat acromion may violate the deltoid origin with subsequent iatrogenic avulsion.

The clinical relevance of post-arthroscopic injury to the anterior acromial attachment of the deltoid origin and its consequent debilitating effect on deltoid muscle strength has not been investigated. Our study aimed to address the impact of arthroscopic acromioplasty on abduction strength and to determine whether acromial anatomy contributes to any potential effect. The null hypothesis was that arthroscopic acromioplasty in acromions without spurring leads to a loss in the abduction strength of the affected shoulder.

## Materials and methods

Consecutive adult patients suffering from subacromial impingement syndrome admitted to the orthopedic outpatient clinic for arthroscopic acromioplasty between 2018 and 2022 were enrolled in the study. The study was approved by the Ethics Committee of Memorial Bahçelievler Hospital (approval no: 106), and informed written consent was obtained from all patients. The included patients with Bigliani type 2 or type 3 acromion did not respond to at least six months of conservative treatment or physical therapy. Patients with rotator cuff tears, calcifying bursitis and tendonitis, osteoarthritis of the glenohumeral joint, adhesive capsulitis, os acromiale, shoulder instability, symptomatic acromioclavicular joint, cervical spine, or any neuromuscular disorder were excluded. The included patients were grouped into two groups according to their acromion morphology: Bigliani type 2 with a flat acromion and Bigliani type 3 with a hooked acromion [2].

A total of 87 patients were initially enrolled in the study, but only 74 were able to meet the study criteria and were ultimately included. The study included 33 patients with a Bigliani type 2 acromion (14 males and 19 females) and 41 patients with a Bigliani type 3 acromion (25 males and 16 females). The mean age of patients in the Bigliani type 2 group at the time of arthroscopic acromioplasty was 49 years and two months, with a range of 31 years and six months to 56 years and 10 months. In contrast, the mean age of patients in the Bigliani type 3 group was 52 years and one month, ranging from 65 years and three months to 36 years and six months. Despite the comparable ages between the two groups (p = 0.92), the limited sample size prevented us from pairing participants based on age and sex.

The diagnosis of isolated impingement syndrome was made by physical examination and subacromial local anesthesia injection test (Neer impingement test) and was confirmed by diagnostic imaging. Both conventional radiographs (including anteroposterior, lateral, and outlet views) and MRI of the affected shoulder were used to evaluate the morphology of the acromion and exclude any other prementioned conditions. The radiological evaluation of acromial morphology via X-ray was performed by two board-certified orthopedic surgeons. 

The surgical intervention comprises initial diagnostic arthroscopy of the glenohumeral joint via the posterior port, followed by subacromial space placement of the arthroscope and thorough bursectomy. Then, arthroscopic assessments of the acromion and bursal-sided rotator cuff were performed. The acromion was graded as either a slightly concave or a hooked acromial undersurface. The extent of coracoacromial ligament recession was limited to the curved undersurface that was planned to be resected, and arthroscopic acromioplasty was conducted by a motorized burr until a flat acromial undersurface was obtained using a cutting block technique [11]. Postoperative radiography was used to confirm the results of the acromioplasty.

**Figure 1:**
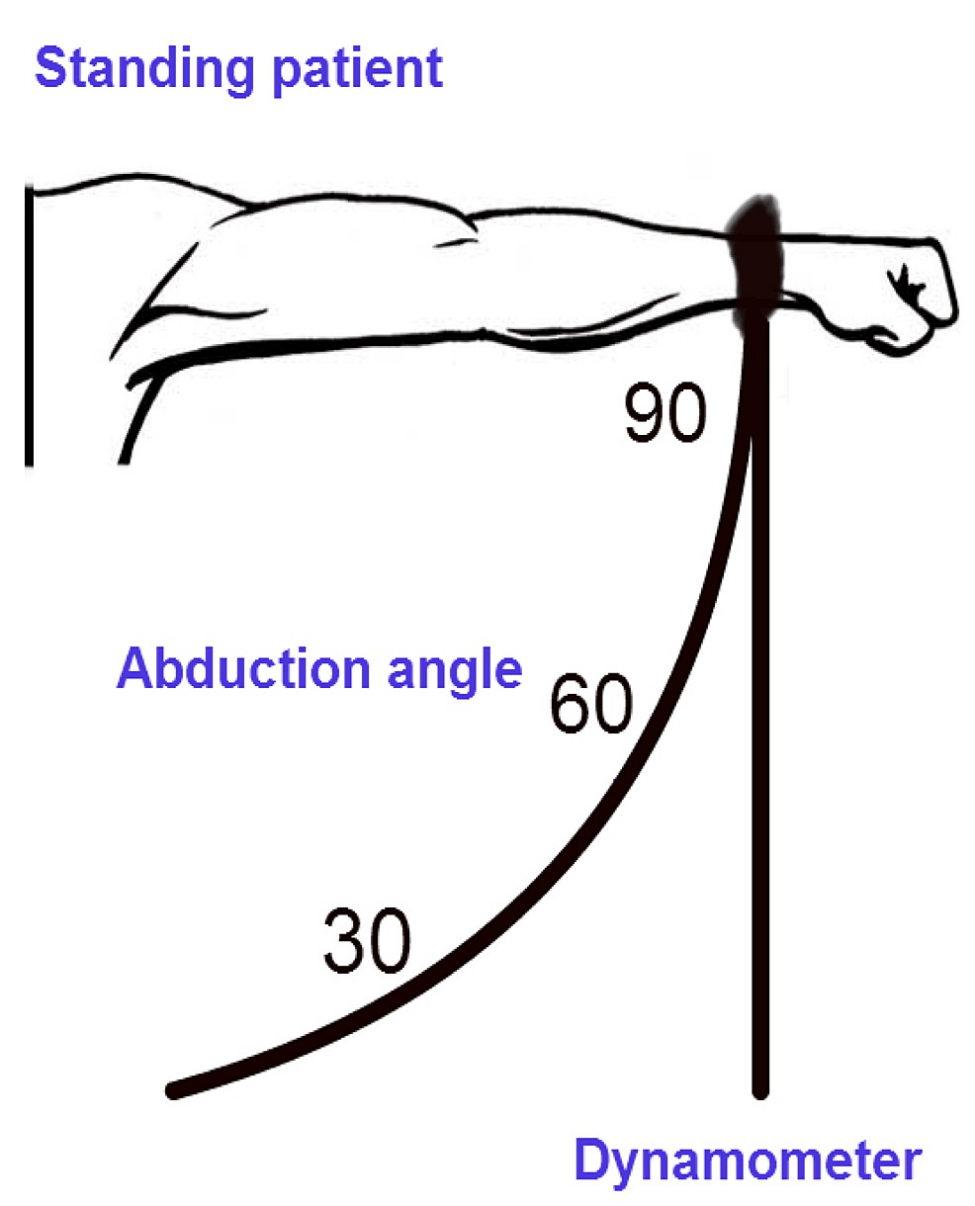
Schematic representation of the abduction strength test.

The abduction strength of the patients was examined using a well-established handheld dynamometer (Isobex®; Cursor AG, Berne, Switzerland) [12]. Following the provision of instructions regarding the testing procedures, we conducted trial sessions and subsequently assessed the subjects' ability to perform maximal isometric contractions for each muscle group, allowing for a three-minute interval between attempts. Measurements of the isometric strength of the shoulder muscles were obtained preoperatively (at 30°, 60°, and 90° abduction in the scapular plane) and at the first, third, and sixth months postoperatively (Figure 1).

Statistical analysis

The data were entered into an Excel spreadsheet (Microsoft® Corp., Redmond, WA). We used t-tests to compare the mean values of strength measurements at different positions. We used Statistical Product and Service Solutions (SPSS, version 13.0; IBM SPSS Statistics, Chicago, IL) for all analyses, and p < 0.05 indicated statistical significance.

## Results

The results revealed a substantial decrease in the abduction strength in both groups at 30°, 60°, and 90° abduction one month after surgery compared to the preoperative values (<0.001).

In the Bigliani type 3 group, at 30° abduction, the mean abduction strength was 6.82 kg preoperatively, 5.13 kg in the first month, 6.12 kg in the third month, and 6.91 kg in the sixth month. Despite the improvement in abduction strength in the sixth month, there was no statistically significant difference between the preoperative and sixth-month values (p = 0.78). Similarly, at 60° and 90° abduction, the abduction strength returned to near-preoperative values in the third month.

In the Bigliani type 2 group, the significant decrease in abduction strength persisted in the third and sixth months. According to the preoperative measurements, the mean abduction strengths for 30°, 60°, and 90° abduction were 8.23 kg, 6.57 kg, and 6.1 kg, respectively. These values decreased to 6.0 kg (p = 0.047), 5.151 kg (p = 0.025), and 4.56 kg (p = 0.006), respectively. These changes were statistically significant. The results are summarized in Table 1.

**Table 1:** Comparison of mean isometric abduction strength.

		Mean strength (kg) ± standard deviation (SD)						
Acromion type	Abduction degree	Preoperative	First month	P value	Third month	P value	Sixth month	P value
Bigliani 2	90°	6.107±2.39	2.604±1.22	<0.001	3.370±1.33	0.003	4.567±1.83	0.006
	60°	6.578±3.18	2.944±1.40	<0.001	3.905±1.71	0.011	5.151±2.37	0.025
	30°	8.23±2.96	3.549±1.35	<0.001	4.598±2.12	0.028	6.004±2.74	0.047
Bigliani 3	90°	4.505±1.95	2.923±1.43	<0.001	3.756±1.78	0.13	4.213±2.10	0.32
	60°	5.130±2.08	4.084±1.82	<0.001	4.746±2.20	0.23	4.987±2.33	0.40
	30°	6.820±3.04	5.136±2.01	<0.001	6.129±3.00	0.67	6.919±3.15	0.780

## Discussion

In the present study, we investigated the effect of arthroscopic subacromial decompression on abduction strength and whether some acromions were more likely to be involved in this effect. Our results showed that although the postoperative abduction strength in both the Bigliani type 2 and type 3 groups was reduced in the first month, patients with the Bigliani type 3 acromion regained their preoperative abduction strength in the third month. However, such recovery was still not evident in patients with the Bigliani type 3 acromion at the sixth month. This finding might be attributed to the detachment of the deltoid originating from the anterior and lateral aspects of the acromion, which might be more pronounced in patients with a Bigliani type 2 acromion.

Evidence of possible deltoid origin detachment during arthroscopic acromioplasty comes from studies by Torpey et al. [9] and Green et al. [10]. These authors showed that in their cadaver's simulated arthroscopic subacromial decompression, a significant portion of the deltoid muscle’s acromial origin was detached during this procedure (up to 80%). Furthermore, in their cadaveric study, Green et al. [10] reported a correlation between the devastating effect of acromioplasty on the deltoid origin and the morphology of the acromion, but a clinical investigation of this correlation was not conducted. The results of our study not only confirm the association between arthroscopic acromioplasty and abduction strength loss but also show that a specific acromion morphology predisposes patients to this loss.

**Figure 2:**
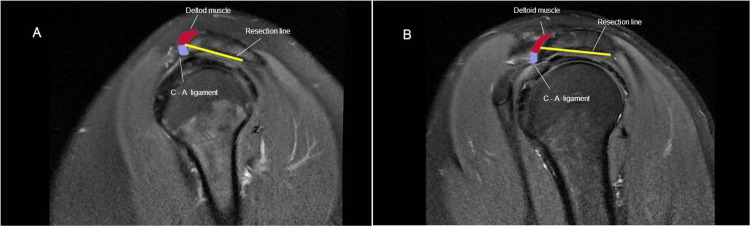
Resection lines and proximity to the deltoid origin in the cutting block technique: (A) Type 3 acromion and (B) Type 2 acromion.

The preservation of abduction strength following hook-shaped (Bigliani type 3) acromioplasty could be attributed to the spurring part of the acromion being devoid of deltoid muscle origin (Figure 2A). Conversely, removing the undersurface of a less-curved acromion (Bigliani type 2) would violate the acromial attachment of the deltoid fibers and consequently affect abduction strength (Figure 2B).

**Figure 3:**
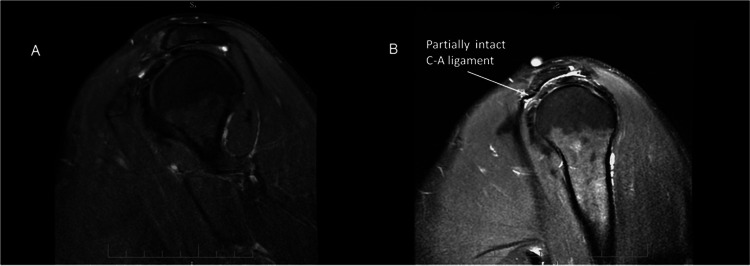
(A) Preoperative MRIs of a patient with type 2 acromion; (B) Postoperative MRI of the same patient showing a partially intact coracoacromial ligament.

Postoperative MRIs of some patients with type 3 acromions, requested for other reasons demonstrated the presence of an intact portion of the coracoacromial ligament, which indicates that the deltoid origin was not substantially affected by acromioplasty (Figures 3A-3B).

Our study demonstrated that decreased abduction strength in patients with Bigliani type 2 acromions following arthroscopic acromioplasty could be especially important for athletes with high performance expectations and workloads. Moreover, a recent multicenter randomized placebo surgery-controlled trial reported that arthroscopic subacromial compression provides no significant medium to long-term superiority over diagnostic arthroscopy in patients with impingement syndrome [13]. Therefore, the surgical indication for arthroscopic acromioplasty should be carefully considered in this group of patients, and a thorough evaluation of the cause of secondary impingement should be excluded before the surgical decision. 

Patients with rotator cuff tears are more dependent on deltoid function; hence, detachment of the deltoid origin during repair increases the risk of loss of function. Goldberg et al. were the first to suggest that acromioplasty was not necessary for the successful repair of full-thickness rotator cuff tears [14]. A recent prospective randomized clinical trial revealed no difference in long-term outcomes between patients who underwent rotator cuff repair with or without acromioplasty [15]. Therefore, improved clinical outcomes could be achieved without impairing the coracoacromial ligament or detaching the deltoid attachment. Further studies showed that the release of the coracoacromial ligament may lead to an increase in glenohumeral instability [16].

One of the most important limitations of the study was the interobserver reliability of the Bigliani classification system, which is regarded as poor to fair at most [2]. The minimization of radiographic grading errors was attempted by relying on three independent blinded radiographic evaluations, and any discrepancies were resolved by accepting the judgment of the majority preoperatively and verified by arthroscopic assessment during acromioplasty. Another limitation of the study was the lack of postoperative MRI data for deltoid origin damage as the clinical course of the patients did not warrant further investigation.

## Conclusions

In conclusion, the findings of the present study demonstrated that arthroscopic acromioplasty may cause decreased abduction strength in patients with type 2 acromions, and acromioplasty should be the primary treatment for type 3 acromions with severe spurring. All secondary impingement pathologies for patients with type 2 acromions should be ruled out before performing arthroscopic subacromial decompression. Otherwise, a decrease in abduction strength is more likely to occur.
